# Physical inactivity level and lipid profile in traditional communities in the Legal Amazon: a cross-sectional study: Physical inactivity level in the Legal Amazon

**DOI:** 10.1186/s12889-022-12973-9

**Published:** 2022-03-18

**Authors:** Erika da Silva Maciel, Bhárbara Karolline Rodrigues Silva, Francisco Winter dos Santos Figueiredo, André Pontes-Silva, Fernando Rodrigues Peixoto Quaresma, Fernando Adami, Fernando Luiz Affonso Fonseca

**Affiliations:** 1 Universidade Federal do Tocantins (UFT), Palmas, TO Brazil; 2 Centro Universitário FMABC (FMABC), Santo André, SP Brazil; 3 Programa de Pós-Graduação em Fisioterapia (PhD Program), Universidade Federal de São Carlos (UFSCar), Rod. Washington Luiz, Km 235, São Carlos, SP CEP 13565-905 Brazil; 4 Universidade Federal de São Paulo (UNIFESP), São Paulo, Brazil

**Keywords:** Exercise, Vulnerable populations, Cholesterol, Physical activity, Public health

## Abstract

**Background:**

The practice of regular physical activity can alter the lipid profile in populations according to diverse demographic characteristics.

**Objective:**

To evaluate the association of physical inactivity with the lipid profile among vulnerable populations.

**Methods:**

A cross-sectional study was conducted among 349 vulnerable individuals from Tocantins state, northern Brazil. The International Physical Activity Questionnaire 7 Day short form was used to measure self-reported physical activity levels. Venous blood samples were drawn to evaluate lipid profile. Logistic regression adjusted by the socioeconomic variables was used to analyse the effects of physical inactivity on the lipidic profile. The level of significance was 5% and Stata® (StataCorp, LC) version 11 was used.

**Results:**

We observe an inverse relationship between physical inactivity and HDL-C—that is, those who were sedentary or below the WHO Recommendations for physical activity were at 2.6 greater odds (IC95% 1.21, 5.67; *p* = 0.015) of having a lower HDL compared with those meeting or exceeding WHO physical activity recommendations.

**Conclusion:**

On the vulnerable populations studied, the insufficiently active or sedentary individuals (called the physical inactivity individuals) have more risk of the altered HDL-C.

## Introduction

The lifestyle and the lipid profile are factors directly involved in the risk of cardiometabolic diseases. However, understanding the lipid profile, their subclasses, and possible associations with lifestyle has been a great challenge in reason of the genetics and socioeconomics features [1]. The most common lipids reported are low-density lipoprotein cholesterol (LDL-C), high-density lipoprotein cholesterol (HDL-C), and triglycerides [2].

High levels of LDL cholesterol increase the risk of cardiovascular complications. The HDL cholesterol carries lipids back to the liver to recycle and disposal; as consequence, high levels of HDL cholesterol indicate a healthier cardiovascular system. Furthermore, HDL cholesterol has other physiological functions as an antioxidant, anti-inflammatory, and antithrombotic activities [1–3].

There is a direct relation between dyslipidemia and coronary diseases, and a reduction of total cholesterol is considered the best treatment for cardiovascular prevention [2]. This is confirmed by the projections of death by cardiovascular diseases in the world, estimated at 16.7 million in 2002 to 23.3 million in 2030, and the prevalence of ischemic cardiac disease which is expected to become the most important cause of deaths in low-income countries by 2030 [4].

Otherwise, physical activity has been shown the best factor of protection related to lifestyle. Regular physical activity changes the lipidic profile, as observed in studies with different populations with different demographical characteristics [2, 3, 5–8], and promotes positive adaptations in the distributions of subclasses of HDL-C and the functions of HDL-C in obese women [3]. Furthermore, the interventions with physical activity (aerobic/ resistant) reduce risk factors of cardiovascular diseases (fat mass, blood pressure, total cholesterol, LDL-C cholesterol), and increase HDL cholesterol [3, 5, 7, 9]**.**

Physical activity in sports and recreation was also positively associated with HDL in the Brazilian elderly [5], however, the frequency, intensity, and duration of the exercise needed to increase the levels of cholesterol are not clearly identified [2]. If both factors (physical activity and HDL) be considered, a household vigilance study from England (between the years of 1998 and 2008) shows that if all the 37.059 participants had adhered to the physical activity guideline, would be a reduction of 4.1 new cases of deaths (cardiovascular disease mortality) in 100 habitants—that reduction represents 68.2% of the incidence in the population; also has been calculated that if all the people had a normal HDL-C, would have a reduction of 0.4 new cases of deaths in 100 habitants, that reduction represents a reduction of 6.6% of the incidence in the population [7].

Adults with cardiometabolic diseases, and low levels of physical activity, are considered risk groups for various health complications (the most recent example is COVID-19) [10]; the lifestyle of the Brazilian population contributes, among other factors, to the high prevalence of cardiometabolic diseases (e.g., diabetes mellitus and cardiovascular diseases), and this has a positive correlation with a high mortality rate [11, 12]. However, to the best of our knowledge, adults from the Legal Amazon communities were not included in the epidemiological studies that evaluated the population’s level of physical activity and lipid profile; either a systematic review (Cochrane, PROSPERO, and PubMed) or a primary study.

Few studies have approached the risk of the not transmissible chronic diseases in traditional communities such (as quilombolas and artisanal fisheries). In quilombolas communities in northeast Brazil, low levels of physical activity were prevalent and have been associated with articular pain, high blood pressure, and overweight [13], in adults and children [14]. In Alagoas (Brazil), a comparative study between quilombola and not quilombola women identified that the quilombola’s have a higher risk to have abdominal obesity and high blood pressure [15]. In artisanal fisheries, although the labor activity has high caloric expenditure because of the manual work, the practice of a physical activity is limited to the labor activities [16]. The artisanal fisheries are characterized by low levels of schooling and by social vulnerability [17, 18].

Evidence suggests that economically inferior groups have lower levels of physical activity, because of the lack of resources, unfavorable environments, and low educational level [14, 19], in this way that factor hypothetically contributes to altered lipidic profiles. In this context we raise the question: What is the impact of physical (in)activity in the lipidic profile of vulnerable populations? This study aims to evaluate the association of physical inactivity with the lipid profile among vulnerable populations.

## Methods

### Study design

A cross-sectional study presented according to the STROBE guidelines [20] in quilombolas (afro-descendant communities) and colonies of artisanal fisheries, in the period of 2015 to 2017, Tocantins’ state, Brazil.

### Settings

The study was made in 5 quilombolas’ communities named: Barra do Aroeira (Santa Tereza County), Morro de São João (Santa Rosa of Tocantins County), Malhadinha, Córrego Fundo, and Manoel João (Brejinho of Nazaré county); and 4 colonies of artisanal fisheries in the state of Tocantins named: Itaobi (Brejinho de Nazaré), Z-10, Associação Parque Sucupira (Palmas), and COPEMITO (Miracema of Tocantins counties). The colonies surveyed are located around the watershed of the Tocantins River, the second largest river in Brazil, with 172.828 km^2^ and of great significance for the state’s economy.

According to data from the Information System on Quilombola Communities in Brazil, there are about 2394 Quilombola communities in the country. The State of Tocantins (Brazil) has 37 recognized quilombola communities, being the sixth place, among the states of Brazil, with more quilombola communities. The number of families residing in each community is estimated, the exact number of residents fluctuates due to inconsistency in cadastral updating and migration to other cities (urban areas). In the municipality of Brejinho de Nazaré there are approximately 90 families in three quilombola communities (Malhadinha, Córrego Fundo, Manoel João) in Santa Rosa do Tocantins approximately 115 families, and in the municipality of Lagoa do Tocantins 20 families during the study.

Among the quilombolas, 79% are users of social programs of the income distribution, nearly 25% do not know to read, almost 75% are in situations of extreme poverty and more than 80% live in the function of agriculture, extractivist and artisanal fishing [21]; Tocantins has 36 colonies of fisheries registered in the Institute of Rural Development of the State of Tocantins (RURALTINS). The searched colonies are located around the hydrographic basin of Tocantins River, the second largest river of Brazil with 172,828 km^2^, which has great significance for the economy of the state because of the fisher and recreation activities there practiced river [18].

The artisanal fishing practiced by the colonies around the Tocantins River is manifested through costumes and sociocultural traditions performed in little boats and low-power equipment. Artisanal fishing has great socioeconomic importance for several countries, such China, India, Japan, and Chile, because represents a source of employment, diversity, and alimentary security [21]. All procedures of this study were previously approved by the ethics committee in research with human beings of a university in Brazil., attending the resolution 466/2012 (opinion number 3219733, and 3.358.190).

### Participants

All the residents from the quilombolas communities and from the colonies of artisanal fishermen of the north of Brazil, in Tocantins, were voluntarily invited to participate in a meeting where are introduced to the research. This community has about 417 adults between 18 and 59 years old and 92 seniors with more than 60 years old. In this amount, nearly 298 resides in quilombolas’ communities and 211 reside in fishermen colonies. Were included all the adults and seniors (*n* = 509) accepted the Free and Informed Consent Term and conclude all the phases of data collection (*n* = 349).

### Variables

The physical (in)activity was used as an exposure variable and the variables about lipidic profile were used as outcome variables. To perform the analyses, on this study we considered quantitative variables, stratified into groups as shown in the chart below (Table 1).

**Table 1:** Variables and classifications

Variables	Classification
Sex	Male
	Female
Age group	Adults
	Elderly
Communities	Fisheries
	Quilombolas
Socioeconomic classification	Class A
	Class B1
	Class B2
	Class C1
	Class C2
	Class D-E
Physical inactivity	No (Very active /Active)
	Yes (Insufficiently active/Sedentary)
Hypertension	Yes
	No
Excess body weight	Yes
	No
Abdominal perimeter	Desirable
	Changed
	Lipidic profile
Non-HDL Cholesterol	Desirable
Changed	
HDL – Cholesterol	
LDL – Cholesterol	
Total – Cholesterol	

### Sociodemographic characteristics

The socioeconomic and demographic characteristics were obtained by a data collection form made by the authors and composed of the variables: age, sex, socioeconomic classification. The socio-economic classification was assessed by the Brazilian Association of Companies of Search Association. The classification was performed in accordance with the criteria of Classification of Economics from Brazil (Class A, B, C, and D-E) [22].

### Body composition

To measure the height and weight variables was used a drywall stamper 206 and a health-o-meter. With this, the Body Mass Index of the participants was calculated according to de World Health Organization [23]; the abdominal measurement was collected in the largest perimeter of the abdomen between the last rib and the iliac crest, following the recommendation of the Brazilian Association to Study of Obesity and Metabolic Syndrome. The World Health Organization establishes a cut-off point increased for cardiovascular risk if the abdominal perimeter is ≥94 cm in men and ≥ 80 cm in women [23].

### Systemic blood pressure

To verify the systemic arterial hypertension was considered the average (of two measures) of systolic and/or diastolic arterial pressure on percentile ≥95 to age and sex, adjusted for the height percentile. Was used a mercury manometer of the Tycos brand, with clamps with three different dimensions (adult, teenager, child) and a pediatric stethoscope of the Littman brand. All the measures were collected by the same researchers (nursing academics), after specific training. The methodological of VII Brazilian Guideline about Hypertension was used [24].

### Biochemical analysis of blood

Were collected by vein puncture and after fasting between 8 and 10 h and 112 h maximum, 5 mL of blood in a dry tube, to dose glucose by colorimetric enzymatic method, and to dose HDL-C by the colorimetric enzymatic method with fully automated spectrophotometer reading. The fraction of non-HDL cholesterol is used to estimate the total number of atherogenic particles on plasma (VLDL + IDL + LDL) and refers to apo B levels too. The non-HDL cholesterol is easily calculated by subtraction of HDL-C from Total Cholesterol: Non-HDL cholesterol = Total Cholesterol – HDL. The non-HDL cholesterol can get a better estimate of the risk in comparison to LDL-C, especially in cases of hypertriglyceridemia associated with diabetes, metabolic syndrome, or renal disease [25].

### Physical activity

We classified the Physical Activity Level—“very active or active” and “insufficiently active or sedentary”—according to the study by Matsudo et al. [26], through the international physical activity questionnaire, short-form (adapted for the Brazilian population)^;^ this measurement of the level of physical activity is internationally used in epidemiological studies that evaluate associations of physical activity with physical and mental health outcomes in adults, however, it is worth mentioning that occupational physical activity (any human activity based on body movement and expenditure energy) is different from exercise (the latter has planning, repetition, well-defined rules and, many times, a proper place for its practice), and this has not been considered (or clarified) in studies [27, 28].

### Bias

To decrease the risk of bias, there was a previous training of application of the international questionnaire of physical activity and about the data of sociodemographic information. An electronic form of data collection was made in Epi info 7.2 to construct the database in duplicates and filled in by two researchers. In the case where there was a divergence of data, a third researcher was consulted. It should be noted that the collection of Physical Activity Level data through a questionnaire is an indirect measure and, therefore, may contain natural measurement errors.

### Study size

Based on initial studies on this population, the sample becomes representative when each group is composed of a number ≥ 100 participants [14, 29]; thus, the study used a convenience sample. This technique was adopted because there are not many studies on this population and, in addition, access to carrying out studies is restricted by factors such as limited access to the research site, structure for in loco data collection, and availability of participants.

### Quantitative variables

To determine abdominal obesity was established as a cut-off point to metabolic syndrome an abdominal perimeter equal or greater to 94 cm in men and 80 cm in women (presence of abdominal obesity), being used the criteria for classification in Euripides’ because it is sample composed by Afrodescendents. The High Density Lipoprotein (HDL) cholesterol parameter used is: desired HDL ≥ 40 mg/dL for men and ≥ 50 mg/dL for women; Low-Density Lipoprotein (LDL): desirable < 160 mg/dL; total cholesterol: desirable < 90 mg/dL [23].

To define Arterial Hypertension the value considered was: ≥ 130 mmHg for systolic or diastolic ≥85 mmHg and for Body Mass Index was used as parameter > 25 Kg/M^2^ for adults and > 27 Kg/M^2^ for seniors (equal or greater to 60 years old accept for Health Ministry) being analysed as the presence or not of overweight, according to standards of World Health Organization. Physical inactivity was considered positive among sedentary and insufficiently active and negative among active and very active, according to criteria of classification proposed by the Guidelines for Data Processing and Analysis of the International Physical Activity Questionnaire.

### Statistical methods

The qualitative variables were described by absolute frequency, relative, and Odds. To analyse the associations of socioeconomic and demographic characteristics with the physical inactivity prevalence were used logistic regression was with the corresponding confidence interval (95% CI). To estimate the association between physical inactivity and variables of the lipidic profile were used adjusted logistic regression by the socioeconomic variable, according to the selection criteria of variables, being *p* = 0.2 to include in the template, and *p* = 0.5 to exclude of the template. For all analyses, the level of significance was 5%. The software used were Stata® (Stata Corp, L.C) version 15.0.

## Results

### Participants

**Fig. 1:**
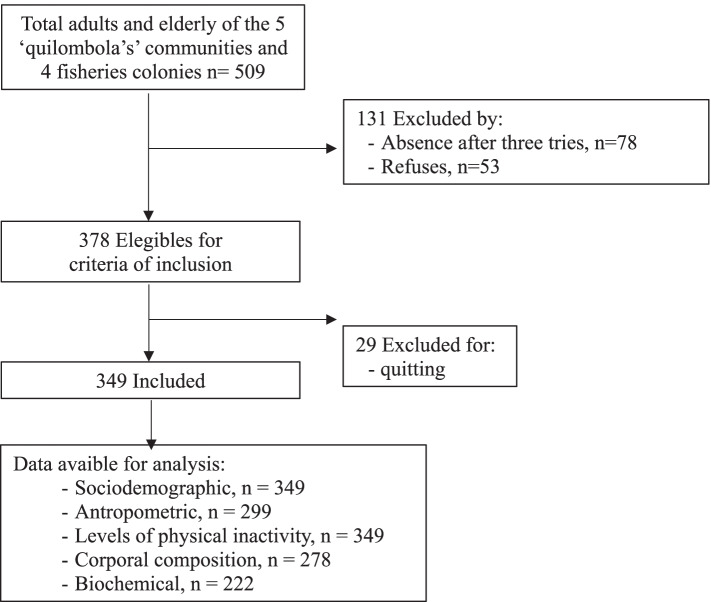
Flowchart of phases of the study

Among the 509 individuals eligible for the inclusion criteria of the five quilombolas’ communities and four fishermen colonies, 349 participate. Four participants were excluded, considered losses for absence after three tries of collection (15%), refuses (10%), and quitting (6%) in the phases. Fig. 1).

### Descriptive data

Were studied 349 persons in vulnerability situation, where the majority were adults (*n* = 282, 81.5%), male (*n* = 181; 51.9%), from economics classes D-E (*n* = 188; 53.9%) and level of physical activity very active or active (*n* = 280; 80.2%). Hypertense individuals prevailed (*n* = 179; 50.4%), and individuals with alteration of abdominal perimeter (*n* = 243; 69.9%) (Table 2). About the lipid profile, most individuals presented non-HDL cholesterol changed (*n* = 122; 58.7%), HDL changed (*n* = 198; 56.1%) and total cholesterol above the recommended levels (*n* = 172; 61.9%) (Table 3).

**Table 2:** Clinical and demographic characteristics according to the sample (*n* = 349)

Characteristics	Values
Sex (male) ^a^	181 (51.9%)
Age range ^a^	
Adult	282 (81.5%)
Elderly	67 (18.5%)
Age (years) ^b^	48.5 (14.3)
Stature (m) ^b^	160.5 (9.6)
body mass (kg) ^b^	68.7 (14.7)
Body Mass Index (kg/m^2^) ^b^	25.0 (3.0)
Abdominal (cm) ^b^	92.0 (12.5)
Hypertension (yes) ^a^	179 (50.4%)
Systole (mmHg) ^b^	124.2 (16.8)
Diastole (mmHg) ^b^	80.0 (11.8)
Overweight (no) ^a^	177 (50.2%)
Abdominal Perimeter ^a^	
Desirable	106 (30.4%)
Changed	243 (69.6%)
Population ^a^	
Fisheries	144 (41.3%)
Quilombolas	205 (58.7%)
Socioeconomical classification ^a^	
Class A	4 (1.1%)
Class B1	3 (0.9%)
Class B2	25 (7.2%)
Class C1	43 (12.3%)
Class C2	86 (24.6%)
Class D-E	188 (53.9%)
Physical Inactivity ^a^	
No (Very active/Active)	280 (80.2%)
Yes (Insufficiently active/Sedentary)	69 (19.8%)

^a^ Values shown as number (percentage); ^b^ Values shown as mean (standard deviation)

**Table 3:** Prevalence of altered levels of non-HDL, HDL, LDL and total cholesterol

Variables	n	(%)
Non-HDL		
Desirable	86	41.3
Changed	122	58.7
HDL		
Desirable	155	43.9
Changed	198	56.1
LDL		
Desirable	247	87.6
Changed	35	12.4
Cholesterol total		
Desirable	106	38.1
Changed	172	61.9

*HDL* High-Density Lipoprotein, desirable HDL - ≥ 40 mg/dL for men and ≥ 50 mg/dL for women, *LDL* Low-Density Lipoprotein, desirable LDL < 160 mg/dL, Cholesterol total: desirable < 190

Tables 2 and 3 consolidate the principal demographic, lifestyle, corporal composition, and biochemical characteristics. In Table 4, it can be observed that socioeconomic and demographic characteristics, as well as lipid profile, had no statistically significant association with physical (in)activity. When analyzing lipid levels associated no physical inactivity, with no statistically significant association was observed, except for HDL-C (Table 5).

**Table 4:** Factors associated to physical inactivity in vulnerable communities

Characteristics	Physical inactivity (n)	Logistic regression		*p*-value
		Odds	OR (CI 95%)	
Sex				
Female	29	0.20		
Male	40	0.28	1.35 (0.79; 2.31)	0.258
Age range				
Adults	48	0.21		
Elderlies	17	0.36	1.69 (0.89; 3.20)	0.104
Communities				
Quilombolas	41	0.24		
Fisheries	30	0.26	1.06 (0.63; 1.80)	0.822
D/E Socioeconomic classification				
No	15	0.25		
Yes	42	0.28	1.12 (0.58; 2.17)	0.737
Hypertension				
No	27	0.24		
Yes	27	0.23	0.96 (0.53;1.74)	0.906
Overweight				
No	32	0.27		
Yes	23	0.18	0.67(0.37; 1.21)	0.190
Abdominal perimeter				
Desirable	20	0.28		
Changed	35	0.20	0.72(0.39; 1.32)	0.291

*OR* Odds Ratio, *95 CI%* 95% Confidence Interval, *Ref* Reference of category

**Table 5:** Physical inactivity associated to non-HDL, HDL, LDL and total cholesterol levels

Physical Inactivity	Number of cases	Logistic regression		*p*	Adjusted Logistic Regression	*p*
		Odds	OR (IC95%)		Adjusted-OR (IC95%)	
Physical Inactivity	Non-HDL					
No	103	1.63				
Yes	23	1.00	0.61 (0.31; 1.18)	0.143	0.49 (0.22; 1.09) *	0.081*
Physical Inactivity	HDL					
No	153	1.18				
Yes	45	1.73	1.46 (0.85; 2.50)	0.167	2.61 (1.21; 5.67)**	0.015**
Physical Inactivity	LDL					
No	30	0.15				
Yes	5	0.09	0.58 (0.22; 1.57)	0.285	0.72 (0.23; 2.27) ***	0.575***
Physical Inactivity	Total Cholesterol					
No	87	0.64				
Yes	21	0.54	0.84 (90.46; 1.51)	0.554	0.91 (0.48; 1.73) ****	0.778****

*HDL* High-Density Lipoprotein, desirable HDL - ≥ 40 mg/dL for men and ≥ 50 mg/dL for women, *LDL* Low-Density Lipoprotein, desirable LDL < 160 mg/dL, Total Cholesterol: desirable < 190; *Ref* Reference Category, *OR* Odds Ratio, IC 95%: Confidence Interval of 95%, *Adjusted by age range, ** Adjusted by population, age range, sex and overweight, ***Adjusted by population, overweight and economic class D/E, **** Adjusted by population and overweight

After multivariable and adjusted by population, age range, sex, and overweight analysis, it was observed that insufficiently active or sedentary presented better odds to have HDL-C alterations (OR 2.61 [IC95% 1.21, 5.67], *p* = 0.015) (Table 5).

## Discussion

### Main results

We observe an inverse relationship between physical inactivity and HDL-C—that is, those who were sedentary or below the WHO Recommendations for physical activity were at 2.6 greater odds of having a lower HDL compared with those meeting or exceeding WHO physical activity recommendations.

In previous studies, Quaresma et al. [14] and Silva et al. [29] pointed out the impossibility of describing the epidemiological profile of communities in the Legal Amazon and highlighted the importance of studies in this context. This reforms the political, clinical, and scientific relevance of the present study, as it is the first research on the level of physical activity and lipid profile in this population (promoting, also, social representation of these communities).

This type of study with vulnerable populations is difficult due to cultural barriers that make access to research difficult. Problems related to feedback on participants’ health situations after the survey limited the number of surveys and the number of participants studied (also making it difficult to build a discussion in the present study).

### Interpretation

Although the benefits of regular practice of physical activity are globally recognized and capable of promoting immensurable effects on health economics to the population, their practice is not yet part of the largest share of people around the world [30]. Level of physical activity is internationally used in epidemiological studies that assess associations of physical activity with different health outcomes (risk of all-cause and cardiovascular mortality, breast and prostate cancer, fractures, recurrent falls, disability, cognitive decline, dementia, Alzheimer’s disease). disease, and depression), however, physical activity is different from exercise, and this has been a confounding discussion in studies [27, 28].

Among the benefits include the rise of HDL cholesterol; reduction of LDL cholesterol, triglycerides, and arterial pressure; improves glucose-insulin homeostasis in fasting and postprandial; induces and maintains weight loss; improves psychological well-being. It is also likely to reduce inflammation; increase endothelial function, and help to stop smoking [9]. The results of this study that physical inactivity was associated with alteration on HDL-C were previously verified in different populations. In this way seems to have a consensus about the health protector effect of practicing physical activity and the inverse relation is also true [2, 3, 5, 6].

A great amount of physical activity (duration and intensity) is associated more significantly with cardiovascular risks factors, including dyslipidemia [6, 7]. Individuals with more than one cardiovascular risk factor such as hypertension, high cholesterol, or smoking, have a better chance to have cardiovascular diseases. Although exercise had been proved to control individuals’ risk factors, the evidence of its effects on multiple risks remains uncertain, it is necessary to realize high-quality clinical trials that evaluate the effect of exercise in people with high cardiovascular risk [9].

Recently some studies have advocated the non-HDL cholesterol to guide lipid control, however, this should be recommended for those who have hypertriglyceridemia or any cardiometabolic abnormality [3, 8]. The HDL has been studied for exerting antiatherogenic effects through biological mechanisms and has anti-inflammatory, anti-apoptotic, and anti-thrombotic effects in endothelial cells of healthy people, in this way, analyse structure and components of HDL rather than the fraction of HDL cholesterol after application of physical training program can be useful to learn about its effects [31].

However, it should be noted the ethical differences in lipidic profiles and in cardiovascular disease risk documented are attributed, in part, to genetic, socioeconomic, and lifestyle differences [1]. In this way, the lifestyle characteristics of the traditional communities studied lead to believe that, although the basic health problems still have no resolution, the prevalence of chronic conditions has gained high proportions in the traditional rural communities, as well as in urbanized ones.

Thus, it contributes to increasing the central problem of health attention systems, with complete epidemiologic transition in developed countries, a social response of fragmented health, with more attention in acute conditions and to acute events of chronic conditions. This situation is not exclusive to Brazil, rural communities in China were found low HDL associated with high levels of central obesity and corporal composition index [32].

A progressive change in the socio-economic situation and a greater influence of western elevated the problems of evitable cardiovascular diseases, it is estimated that the prevalence of ischemic cardiac disease is the principal cause of death also in countries with low income until 2030 [1]. In the case of artisanal fishermen, despite good economic indicators of the production sector and commercialization, the traditional fishermen have big difficulties to get in other activities and the profit from fishing is not sufficient to have a life with better quality [17].

The quilombolas is yet found in the communities high poverty rates, as well as higher rates of malnutrition, lower rates of sanitation and education of the country; about 74.73% of families, are found in an extreme poverty situation, having as main source income benefits from the government, they are a group with special risk to cardiovascular morbimortality, justified the priority in the implementation of attention measures [33]. The characteristics of both groups there studied to show the necessity to protect, prevent and improve health and education measures, minimal conditions, free and quality to improve their life quality.

The present study has limitations that must be addressed. The first one refers to the design of this research (cross-sectional), which has no explanatory power on cause and effect (thus, we suggest conducting a longitudinal study to provide additional information related to the variables investigated in this study); the second refers to the sampling method (for convenience), because, although the sample size was adequate for the outcome investigated in this study, little is known (in the scientific contingent) about the characteristics studied in vulnerable populations; and the third refers to the lack of nutritional monitoring of the participants—we emphasize that research in these communities is of great complexity, whether due to cultural barriers, logistical barriers to accessing communities, or due to the low investment in research in this context.

## Conclusion

The alteration in lipid profile in relation to physical inactivity in vulnerable populations indicates an increase in risk alteration of the HDL cholesterol. Other risk factors studied (sex, age range, socioeconomic classification, hypertension, overweight, abdominal perimeter, non-HDL cholesterol, LDL cholesterol, and total cholesterol) were not related to the impact of physical inactivity on the lipid profile in a vulnerable population.

## Data Availability

The data that support the findings of this study are available from [email – Corresponding author] but restrictions apply to the availability of these data, which were used under license for the current study, and so are not publicly available. Data are however available from the authors upon reasonable request and with permission of [Corresponding author].

## Abbreviations

HDL: High-Density Lipoprotein
LDL: Low-Density Lipoprotein
